# Ribosome Biogenesis in Archaea

**DOI:** 10.3389/fmicb.2021.686977

**Published:** 2021-07-22

**Authors:** Paola Londei, Sébastien Ferreira-Cerca

**Affiliations:** ^1^Department of Molecular Medicine, University of Rome Sapienza, Rome, Italy; ^2^Biochemistry III – Regensburg Center for Biochemistry, Institute for Biochemistry, Genetics and Microbiology, University of Regensburg, Regensburg, Germany

**Keywords:** archaea, ribosome, ribosome biogenesis, ribosomal RNA, ribosomal proteins, RNA modifications

## Abstract

Making ribosomes is a major cellular process essential for the maintenance of functional ribosome homeostasis and to ensure appropriate gene expression. Strikingly, although ribosomes are universally conserved ribonucleoprotein complexes decoding the genetic information contained in messenger RNAs into proteins, their biogenesis shows an intriguing degree of variability across the tree of life. In this review, we summarize our knowledge on the least understood ribosome biogenesis pathway: the archaeal one. Furthermore, we highlight some evolutionary conserved and divergent molecular features of making ribosomes across the tree of life.

## Ribosomal Subunit Composition: Archaeal Specificity and Common Features

The ribosome is a universally conserved ribonucleoprotein (RNP) complex required for the synthesis of polypeptides from the intermediate molecule carrying the genetic information, the messenger RNA (Melnikov et al., 2012; Bowman et al., 2020). The birth of a ribosome itself is a highly energy-consuming and complicated orchestrated molecular dance that culminates in the formation of translation-competent mature ribosomal subunits (Nomura, 1999; Warner, 1999). The mature ribosome is composed of two ribosomal subunits, the small and large ribosomal subunits (hereafter SSU and LSU, respectively). These ribosomal subunits can be further divided into two main classes of structural components, the ribosomal RNA (rRNA) and the ribosomal proteins (r-protein). Despite its universality, the sequence and composition of the ribosomal subunits’ structural components diverge across and within the different domains of life (Melnikov et al., 2012; Ban et al., 2014; Bowman et al., 2020). Notably, the sequence variabilities seen among the universally conserved ribosome structural components were recognized and harnessed at the end of the 1970s by the pioneering studies of Carl Woese and his collaborators and are still the cornerstone of modern molecular phylogenetic analysis and microbial taxonomy (Fox et al., 1977; Woese and Fox, 1977; Woese et al., 1990; Albers et al., 2013; Bahram et al., 2019).

**FIGURE 1:**
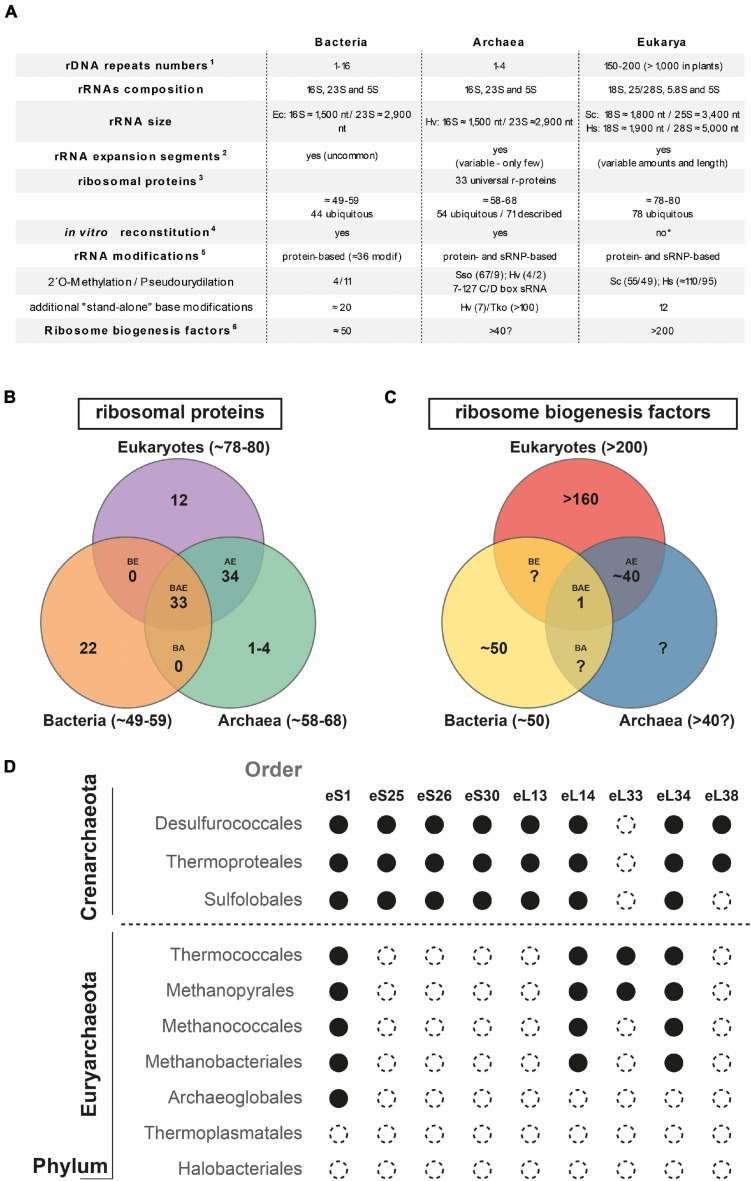
Ribosome and ribosome biogenesis key features overview across the tree of life. **(A)** Summary of ribosome and ribosome biogenesis key features. Modified from Ferreira-Cerca (2017) according to ^1^(Hadjiolov, 1985; Warner, 1999; Klappenbach et al., 2001; Stoddard et al., 2015); ^2^(Gerbi, 1986, 1996; Armache et al., 2013; Parker et al., 2015; Petrov et al., 2015); ^3^(Lecompte et al., 2002; Nakao et al., 2004; Yutin et al., 2012); ^4^(Londei et al., 1986; Sanchez et al., 1990, 1996; Nierhaus, 1991; Mangiarotti and Chiaberge, 1997; Culver, 2003; Nierhaus and Lafontaine, 2004); ^5^(Lafontaine and Tollervey, 1998; Grosjean et al., 2008; Dennis et al., 2015; Sharma and Lafontaine, 2015; Krogh et al., 2016; Sloan et al., 2016; Taoka et al., 2018; Coureux et al., 2020; Grünberger et al., 2020; Sas-Chen et al., 2020); and ^6^(Hage and Tollervey, 2004; Thomson et al., 2013; Woolford and Baserga, 2013; Ebersberger et al., 2014; Grosjean et al., 2014; Henras et al., 2015). The detailed list of putative eukaryotic ribosome biogenesis factors conserved in archaea is depicted in Ebersberger et al. (2014). Abbreviations used: Sso, *Saccharolobus solfataricus*; Hv, *Haloferax volcanii;* Tko, *Thermococcus kodakarensis;* Hs, *Homo sapiens*; Sc, *Saccharomyces cerevisiae*. **(B,C)** Summary of shared ribosomal proteins **(B)** and ribosome biogenesis factors **(C)** across the three domains of life. Numbers of r-proteins and putative ribosome biogenesis factors sequence homologs shared between bacteria, archaea, and eukarya (BAE); bacteria, archaea (BA), archaea and eukarya (AE), bacteria and eukarya (BE), or unique to bacteria (B), or archaea (A), or eukarya (E), are indicated [based on (Lecompte et al., 2002; Hage and Tollervey, 2004; Nakao et al., 2004; Márquez et al., 2011; Yutin et al., 2012; Thomson et al., 2013; Woolford and Baserga, 2013; Ban et al., 2014; Ebersberger et al., 2014; Grosjean et al., 2014; Henras et al., 2015; Coureux et al., 2020; Nürenberg-Goloub et al., 2020)]. **(D)** Exemplary gene distribution of selected archaeal ribosomal proteins shared between archaea and eukaryotes across two major archaeal Phyla. Black circle denotes the presence and open circle denotes the absence of sequence homolog for the indicated ribosomal protein of the small (S) or large (L) ribosomal subunits, respectively. Adapted from Lecompte et al. (2002); Yutin et al. (2012) using the nomenclature proposed in Ban et al. (2014).

Similar to their bacterial counterparts, archaeal ribosomes are composed of three types of rRNAs: the SSU 16S rRNA and the LSU 23S and 5S rRNAs, which interact with 60–70 r-proteins, establishing an intricate macromolecular network (Melnikov et al., 2012; Ban et al., 2014; Bowman et al., 2020; Figure 1).

Up to now and due to the size and sequence similarities among organisms lacking a cell nucleus, the archaeal rRNA molecules have been largely seen as being of a prokaryotic nature (Figure 1). Particularly and in contrast to canonical prokaryotic rRNAs, most eukaryotic rRNAs are characterized by the presence of so-called expansion segments (ES), which are additional RNA elements of various sizes incorporated into the universal prokaryotic rRNA core (Gerbi, 1996; Bowman et al., 2020; Figure 1). These ES increase the size and complexity of the respective rRNAs; however, recent analyses have provided evidence for the presence of such ES in both bacteria and archaea (Armache et al., 2013; Penev et al., 2020; Tirumalai et al., 2020; Stepanov and Fox, 2021). Although most of these sequence additions are limited in size and number (Armache et al., 2013; Penev et al., 2020; Tirumalai et al., 2020; Stepanov and Fox, 2021), larger ES, similar in size to those commonly observed in eukaryotes, have been recently described in the Asgard archaeal phylum (Penev et al., 2020), which is proposed to be the cradle of the eukaryotic lineage (Spang et al., 2015; Zaremba-Niedzwiedzka et al., 2017; Liu Y. et al., 2021). However, a common evolutionary relationship—based on sequence and/or structure homology—of the larger archaeal and eukaryotic ES could not be established (Penev et al., 2020). Recently, a role of some of these ES in ribosomal biogenesis and/or function has been established in eukaryotes (Ramesh and Woolford, 2016; Fujii et al., 2018; Díaz-López et al., 2019; Shankar et al., 2020). Accordingly, determining both the respective function(s) and evolutionary origin(s) of these additional rRNA segments in archaea is of general interest for the field and will be crucial to distinguish between the archaeal origin of eukaryotic features from the independent but convergent evolution trajectories of structural elements present in both archaea and eukaryotes.

The archaeal ribosomal proteins can be divided into three different groups: (1) the universally conserved r-proteins that form, with the rRNAs, the universal ribosomal core (Melnikov et al., 2012), (2) the r-proteins exclusively shared between archaea and eukaryotes, and (3) the archaeal-specific r-proteins (Lecompte et al., 2002; Márquez et al., 2011; Yutin et al., 2012; Ban et al., 2014; Coureux et al., 2020; Nürenberg-Goloub et al., 2020; Figure 1). The absence of exclusively shared r-proteins between bacteria and archaea remains an intriguing observation.

Among the 70 different r-proteins described in archaea, only 54 are known to be ubiquitous across archaea; among them, 33 are universally conserved (Lecompte et al., 2002; Yutin et al., 2012; Ban et al., 2014; Figure 1). The composition variability of the r-protein complement also correlates with a general decrease in complexity of the r-proteins composition at the domain scale (Lecompte et al., 2002; Yutin et al., 2012; Figure 1). In other words, the r-protein counterpart of the last archaeal common ancestor was likely more complex than that of most of its descendent lineages (Lecompte et al., 2002; Yutin et al., 2012). The functional consequences and additional adaptations underlying such r-protein reductive evolution for archaeal ribosome biogenesis and function is currently unknown. Furthermore, recent studies also indicate the presence of archaeal-specific ribosomal proteins (Márquez et al., 2011; Coureux et al., 2020; Nürenberg-Goloub et al., 2020), suggesting that the discovery of new additional archaeal-specific r-proteins is still incomplete. Last, organism-specific insertion, extension, deletion, or sequence variations within conserved r-proteins are not unusual, and may play an important role for the cellular adaptation of ribosome biogenesis and function (Ferreira-Cerca et al., 2007; Melnikov et al., 2018; Dao Duc et al., 2019). However, the functional contributions of the additional archaeal-specific r-protein features for ribosome assembly and function remain to be explored.

Another particularity of the r-protein composition of some archaeal ribosomal subunits is the presence of intra- and inter-subunit promiscuous r-proteins, which leads to an increase of the respective r-protein stoichiometry and to the presence of shared structural components of both the SSU and LSU (Armache et al., 2013). This peculiarity is in stark contrast to what is typically observed in the bacterial and eukaryotic systems, in which r-proteins are thought to be exclusive structural components of one or the other ribosomal subunit present in one copy per ribosomal subunit, with the exception of the LSU stalk r-proteins (Armache et al., 2013). The functional implications of these molecular peculiarities remain to be analyzed.

In conclusion, the core structural components of the archaeal ribosomal subunits are of prokaryotic origin, to which archaeal-specific and shared archaeal-eukaryotic features have been added. Together, the structural and functional constraints and/or advantages of these structural and compositional idiosyncrasies for ribosome biogenesis and function remain to be explored.

## rRNA Organization, Synthesis, and Processing in Archaea

The organization of the rRNA genes and the maturation of the transcripts thereof to yield mature rRNA molecules is the most widely studied and best understood aspect of ribosome biogenesis in archaea (Yip et al., 2013; Ferreira-Cerca, 2017; Clouet-d’Orval et al., 2018). Because a large literature, including a number of excellent reviews, exist on this topic, here only the features most relevant from an evolutionary point of view are described.

**FIGURE 2:**
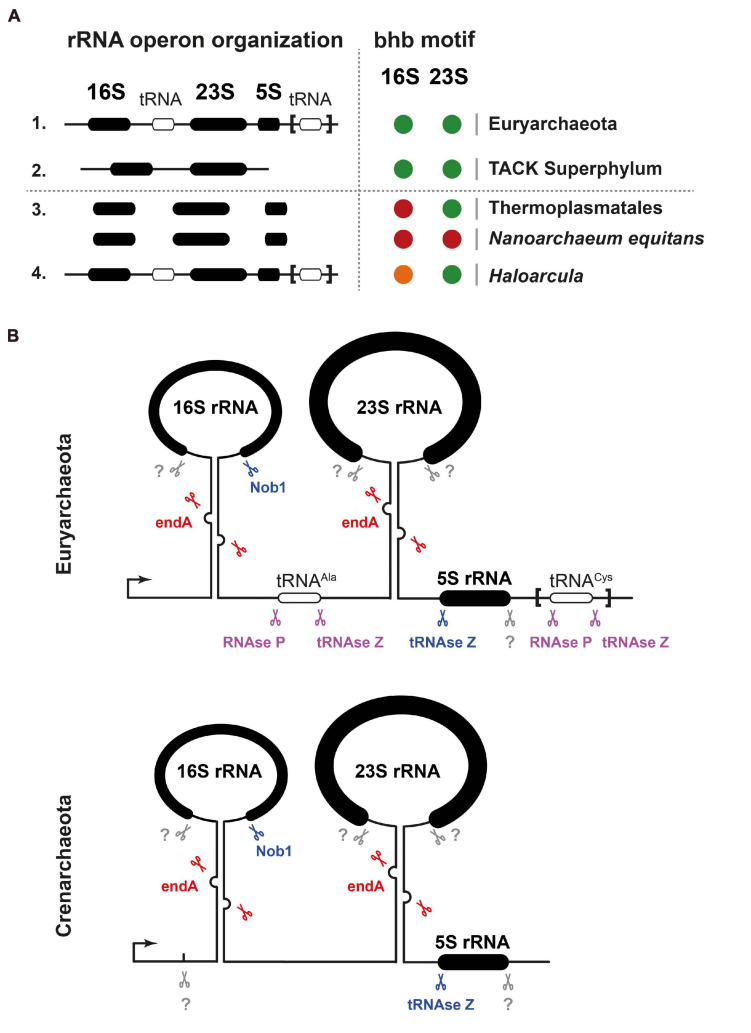
rDNA gene organization and processing of pre-rRNA in archaea. **(A)** Ribosomal DNA gene organization and rRNA BHB motif conservation across archaea. A selected survey of archaeal rRNA operon organizations suggests two predominant classes of linked rRNA organization found in representative organisms of the Euryarchaeota and TACK Superphylum (Thaumarchaeota–Aigarchaeota–Crenarcheota–Korarchaeota) and one minor class of unlinked organization (e.g., *Thermoplasmata* class*/Nanoarchaeum equitans*). 16S and 23S rRNAs processing stem secondary structures were predicted using the ViennaRNA Web servers. Presence of predicted BHB is indicated in black. Presence of heterogeneous rRNA operons with heterogeneous presence of BHB motif within the processing stem is depicted in orange (*Haloarcula* genus). Absence of predictable BHB motifs is depicted by a red circle (e.g., *Thermoplasmata* class*/Nanoarchaeum equitans*). Modified from Jüttner et al. (2020) under CC-BY License. **(B)** Schematic representation of exemplary rRNA processing sites and the known respective ribonuclease activities required for the maturation or the pre-rRNA are indicated. Unknown activities are indicated in gray, putative activities in lilac, activities base on *in vitro* analysis in blue, and activities based on *in vivo* analysis in red. Upper panel represents common organization found in Euryarchaeota and lower panel in Crenarchaeota. Modified from Ferreira-Cerca (2017); Jüttner et al. (2020).

As described, archaeal ribosomes are composed of one 30S and one 50S ribosomal subunit, the former containing a 16S rRNA and the latter 23S and 5S rRNAs. The genomic organization of the rRNA genes, however, presents marked differences in the different archaeal groups. Most euryarchaeota have a typically bacterial operon organization with the 16S-23S-5S rRNA genes linked in this order, separated by spacer sequences, and transcribed all together. In most cases the spacer separating the 16S and the 23S rRNA genes contains an Ala-tRNA gene; some euryarchaea also have a second tRNA gene, Cys-tRNA, in the 3′ETS downstream of the 5S rRNA gene (Figure 2). By contrast, in the crenarchaeota and probably in most members of the TACK superphylum, the 5S rRNA genes are physically separated from the other two larger rRNAs and transcribed independently (Figure 2). There are also a few special situations, such as that of the euryarchaeon *Themoplasma acidophilum*, where the three 16S, 23S, and 5S rRNA genes are unlinked and separately transcribed (Yip et al., 2013; Brewer et al., 2020; Figure 2).

The primary rRNA transcripts are maturated following pathways that follow neither the bacterial nor the eukaryal paradigm, albeit having features reminiscent of both.

As in bacteria, the sequences flanking the rRNA genes have extended complementarity and pair, forming double-helical stems that are the target of certain endonucleases starting rRNA maturation. However, although, in bacteria, these stems are cleaved by RNAse III, in most archaea, they typically contain Bulge-helix-Bulge (BHB) motifs that are recognized and cleaved by the archaeal-specific *endA* splicing endonuclease (Tang et al., 2002; Ferreira-Cerca, 2017; Clouet-d’Orval et al., 2018; Qi et al., 2020; Schwarz et al., 2020; Figure 2). Consequently, the pre-16S and pre-23S rRNAs are ligated and first released in a circular pre-rRNA form, which is subsequently opened and matured by other enzymes that have not yet been characterized (Tang et al., 2002; Ferreira-Cerca, 2017; Clouet-d’Orval et al., 2018; Jüttner et al., 2020; Qi et al., 2020; Schwarz et al., 2020). For a comprehensive review of the rRNA maturating/modifying enzymes, see Clouet-d’Orval et al. (2018) and Ferreira-Cerca (2017).

In certain members of the crenarchaeota, the processing of 16S rRNA has features that present some homology with the eukaryotic process; specifically, there are endonucleases that introduce 1-2 cuts within the 5′ETS (Durovic and Dennis, 1994; Figure 2). The most distal of these processing sites, termed site 0, lies some 70 nucleotides ahead of the 16S mature 5′ end, is probably conserved in most crenarchaeota, and has similarity to the processing site termed A0 in eukaryotes. Site A0 is generally present in eukaryotic pre-rRNAs and is one of the earliest processing sites starting its maturation (Mullineux and Lafontaine, 2012). In archaea, endonucleolytic cleavage at site 0 is independent of the formation of the processing stems containing the BHB motifs. Instead, its recognition is guided by a specific sequence containing a conserved CUU motif also found in the eukaryotic counterpart. This CUU motif is shown to be essential for cleavage in *S. solfataricus* (Ciammaruconi and Londei, 2001). Notably, in the eukarya, cleavage at site A0 requires a RNP particle containing the small nucleolar RNA U3, but in the archaea this does not seem to be the case. The archaeal endonuclease cutting at site 0 has not yet been identified; interestingly, it seems to be closely associated with the 60 kDa chaperonin, at least in *S. solfataricus* (Ruggero et al., 1998).

Although homologs of eukaryotic small nucleolar RNAs do not seem to be involved in rRNA processing in archaea, they do participate massively in another prominent feature of archaeal rRNA maturation, that is, guiding chemical modifications of specific nucleotides, which is described in the next paragraph.

## Ribosomal RNA Modifications

RNA modifications were discovered in the early 1950s, and since then, more than 100 different types of chemical modifications have been described (Littlefield and Dunn, 1958; Boccaletto et al., 2018). These modifications are expanding the chemical and structural properties of the classical RNA alphabet (Li and Mason, 2014; Kadumuri and Janga, 2018).

Ribosomal RNA modifications are found in all rRNAs studied thus far (Piekna-Przybylska et al., 2008; Boccaletto et al., 2018); however, their diversity (respective chemical nature, number, and position) can be diverging across archaea (Grosjean et al., 2008; Dennis et al., 2015; Boccaletto et al., 2018; Coureux et al., 2020; Sas-Chen et al., 2020). rRNA modifications can be grouped into two main types: (1) base and (2) ribose modifications. Furthermore, the machineries involved in the rRNA modification process can be also subdivided into two major groups: (1) stand-alone enzymes, which are found across all domains of life, and (2) RNA-guided modifications, which utilize RNP complexes to guide and modify the target rRNA in an RNA sequence-dependent manner (Lafontaine and Tollervey, 1998; Omer et al., 2003; Yip et al., 2013). Notably, these RNP complexes are ubiquitous in both archaea and eukaryotes but are absent from bacteria and are responsible for the two major types of rRNA modifications, i.e., 2′O-methylation of the ribose moiety by the C/D box sRNPs and isomerization of the uridine base into pseudouridine by the H/ACA box sRNPs (Lafontaine and Tollervey, 1998; Omer et al., 2003; Yip et al., 2013). Moreover, in eukaryotes, few snoRNPs do not have any known rRNA modification function but are instead required for pre-rRNA processing (Lafontaine and Tollervey, 1998; Sharma and Lafontaine, 2015; Sloan et al., 2016). Among these, the snoRNA U3 is required for early processing steps of the SSU and to avoid premature folding of the SSU central pseudoknot structure (Baßler and Hurt, 2019; Klinge and Woolford, 2019). In archaea, U3 and snoRNPs facilitating rRNA processing and folding independently of rRNA modification activity are not known. More details about these two classes of RNPs and their rRNA modifications in archaea can be found in the two accompanying reviews in this special issue by Randau and collaborators (C/D box sRNPs; Breuer et al., 2021) and Kothe and collaborators (H/ACA box sRNPs; Czekay and Kothe, 2021).

In addition to the two main types of rRNA modifications mentioned, additional base modifications are also found. Commonly, base methylations (m1, m3, m5, m6A, …) and also acetylation or larger types of modifications (e.g., acp3) are decorating the rRNAs (Piekna-Przybylska et al., 2008; Boccaletto et al., 2018). Generally, most of these modifications cluster within the ribosomal subunit functional centers (A-, P-, E-sites, and subunit bridges) and are believed to stabilize and/or support the activity of the translation machinery (Piekna-Przybylska et al., 2008; Sharma and Lafontaine, 2015; Sloan et al., 2016). Interestingly, the position and/or chemical nature of these modifications is apparently flexible across the tree of life, suggesting that the functional contribution of the respective rRNA modification(s) in their respective structural environments prevails over their exact chemical nature and/or relative position (Piekna-Przybylska et al., 2008; Sharma and Lafontaine, 2015; Sloan et al., 2016; Ferreira-Cerca, 2017).

**FIGURE 3:**
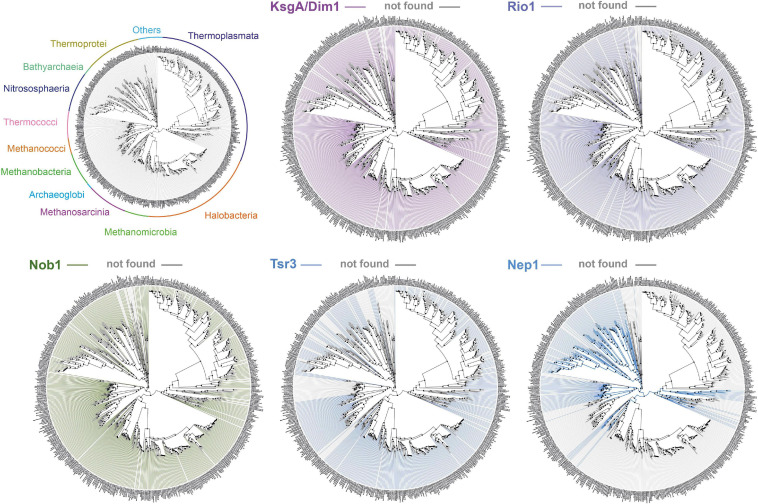
Exemplary conservation of selected putative ribosome biogenesis factors involved in small ribosomal subunit biogenesis in archaea. Phylogenetic conservation profile of the indicated known or putative small ribosomal subunit ribosome biogenesis factors across 1,500 archaeal genomes were generated using AnnoTree (http://annotree.uwaterloo.ca; Mendler et al., 2019). Archaeal classes are annotated in a phylogenetic tree (upper left) as provided by AnnoTree. Absence of sequence homolog in a define organism is indicated by a gray line, whereas its presence is indicated by a colored line. Note the absence of significant homology for Nep1 (e.g., Thermoplasmata, Halobacteria, and more) or Tsr3 (e.g., Thermococcales) in a large group of organisms, in contrast to the more widespread distribution of KsgA/Dim1, Rio1, and Nob1 archaeal homologs.

The total amounts and types of rRNA modifications strongly vary across archaea. For instance, halophilic archaea possess a lower total amount of rRNA modifications (e.g., *H. volcanii* ∼10 known modifications; Grosjean et al., 2008). For example, the archaeal homologs of the eukaryotic methyltransferase Nep1 are not found in the phylogenetically related Methanogen class II and Haloarchaea (see also Figure 3). This decrease in the number of RNA modifications also correlates with a generally reduced amount of r-proteins and ribosome biogenesis factors in these organisms (Lecompte et al., 2002; Yutin et al., 2012; Ebersberger et al., 2014; see above and below). In contrast, the total amount of rRNA modifications in thermophiles and hyperthermophiles is particularly increased (Dennis et al., 2015). For example, representative organisms of the Thermococcales order, which can grow at remarkably high temperatures (near the boiling point of water), contain a large amount of base acetylations, presumably introduced by the archaeal homolog of the eukaryotic RNA cytidine acetyltransferase Kre33/Nat10 (Sleiman and Dragon, 2019; Coureux et al., 2020; Grünberger et al., 2020; Sas-Chen et al., 2020). Moreover, and in contrast to the clustered distribution of rRNA modifications normally observed, these acetylations are scattered throughout the rRNA sequences (Coureux et al., 2020; Grünberger et al., 2020; Sas-Chen et al., 2020). Furthermore, the total amount of these acetylations seems to vary according to the growth temperature (Sas-Chen et al., 2020).

Remarkably, among all the known stand-alone enzymes, the SSU dimethyltransferase KsgA/Dim1 carrying the dimethylation of two universally conserved adenosines at the 3′end of the SSU rRNA is the only almost universally conserved factor involved in ribosome biogenesis (Lafontaine et al., 1994; Connolly et al., 2008; Seistrup et al., 2017; Knüppel et al., 2021). Despite its widespread distribution, several functional aspects of the KsgA/Dim1 biology, such as assembly/release mechanisms and the modification process itself (e.g., completion) strikingly diverge between different organisms and across the different domain of life (Van Buul et al., 1984; Formenoy et al., 1994; Lafontaine et al., 1994; Connolly et al., 2008; Zorbas et al., 2015; Ghalei et al., 2017; Seistrup et al., 2017; Knüppel et al., 2021; Liu K. et al., 2021).

Overall, these observations suggest that the relative amount of rRNA modifications and their diversity may reflect organism-specific adaptation to their respective environmental conditions and/or organism-specific evolutionary trajectories (Dennis et al., 2015; Seistrup et al., 2017; Sas-Chen et al., 2020; Knüppel et al., 2021). The functional significance of the variability in rRNA modifications, of the presence of different modification machineries on the ribosome biogenesis pathway in archaea, and how these machineries have contributed to (re-)shape the ribosome assembly pathway remains to be determined.

## Assembly of Archaeal Ribosomes: *in vitro* Studies

The capability of bacterial ribosomes to assemble spontaneously *in vitro* from the separate RNA and protein components was first demonstrated in the late 60’s by the Nomura laboratory with *Escherichia coli* 30S subunit (Traub and Nomura, 1968) and later by the Nierhaus laboratory with the 50S subunit from the same organism (Nierhaus and Dohme, 1974). Ribosomes from other bacterial species were also successfully reconstituted *in vitro* (Green and Noller, 1999; Agalarov et al., 2016).

These experiments are important in showing that, even in a huge macromolecular complex such as the ribosome, the components contain in themselves all the necessary information to interact in an orderly way so as to form a functional particle. Even more importantly, they highlight a definite assembly hierarchy, in which a subset of ribosomal proteins starts the ribosome biogenesis process by binding directly to specific sites on the rRNA. These “early assembly” proteins, together with the rRNA, create a “core particle” that has to undergo certain conformational changes before binding the missing proteins and being converted into the final functional particle.

Experiments of *in vitro* assembly with purified components, could define an “assembly map,” i.e., the stepwise binding of ribosomal proteins to the rRNAs leading to the formation of intermediate particles that are finally converted into a complete functional ribosomal subunit (Roth and Nierhaus, 1980). However, the necessary experimental conditions (e.g., time, temperature, and ionic strength, etc.) to enable these *in vitro* reconstitution experiments are commonly incompatible with the physiological conditions of the respective organisms, thereby suggesting the presence of facilitating molecular mechanisms *in vivo*.

Among these mechanisms, the “assembly gradient” originally proposed by Knud Nierhaus suggests that cotranscriptional and directional assembly of r-proteins (5′ to 3′ direction), facilitate the initial steps of ribosomal assembly *in vivo* (Nierhaus, 1991). Similar principles of ribosomal assembly seem to apply in some eukaryotes [see, e.g., Cheng et al. (2017); de la Cruz et al. (2015); Ferreira-Cerca et al. (2007), but see also Cheng et al. (2019) and references therein] and may, therefore, likely operate in the archaeal context. For example, our recent work suggests a 5′ to 3′ coordination of the initial pre-rRNA maturation steps in *H. volcanii* (Jüttner et al., 2020). Moreover, recent studies in Sulfolobales suggest local clustering of the rRNA and r-protein operon genes, which may potentially have implication for early steps of ribosome assembly in some archaea (Takemata and Bell, 2021). However, the conservation of the topology and organization of the ribosome synthesis machinery remains to be explored (Cockram et al., 2021; Sobolev et al., 2021).

Furthermore, additional ribosome biogenesis factors facilitating or speeding up ribosome assembly were also identified later (Bunner et al., 2010; Nikolay et al., 2018; see below). Even if the pathways for *in vitro* ribosome assembly are likely to be at least in part different from those adopted *in vivo*, the results from *in vitro* studies reveal that ribosome biogenesis is a highly coordinated process that requires a number of specific sequential steps to be completed successfully.

*In vitro* reconstitution experiments were also employed to explore the degree of conservation of ribosomal components among different bacterial species. It was demonstrated that hybrid, active ribosomes could be successfully reconstituted from proteins and rRNA from different sources, thus further highlighting the high degree of functional and structural conservation of bacterial ribosomes (Higo et al., 1973; Vogel et al., 1984).

That archaeal ribosomes were also capable of spontaneous self-assembly *in vitro* was demonstrated some years later with the particles of two different extremophilic archaea: the 50S subunits of *Saccharolobus* (formerly *Sulfolobus*) *solfataricus* (Londei et al., 1986), an extreme thermophile, and both 30S and 50S subunits of *Haloferax mediterranei*, a halophilic organism (Sanchez et al., 1990, 1996). The challenge here was not only to obtain spontaneous reassembly of the ribosomal particles from a different domain of life, but also to explore how living in extreme environments affected ribosome biogenesis.

The thermophilic archaeon *S. solfataricus* is a particularly interesting case because it thrives optimally at a temperature of 80–85°C and because it is known to have more protein-rich ribosomes than its bacterial counterparts (Schmid and Böck, 1982; Londei et al., 1983). *S. solfataricus* 50S subunits could be functionally reassembled from the separate RNA and protein components only at high temperatures (80°C) and using high polyamine (thermine) concentrations. Interestingly, the best conditions for *Sulfolobus* 50S subunits *in vitro* assembly entailed a two-step procedure such as for the case of the corresponding *E. coli* particles. As in *E. coli*, the first step is performed at a relatively low temperature (60°C) and yields complete but functionally inactive particles. Activation is only achieved upon incubation at temperatures close to the one optimal for *Sulfolobus* growth (85°C), suggesting the requirement for a temperature-driven conformational change. The presence of a high concentration of the polyamine thermine, which is physiologically present in *S. solfataricus*, is most probably required to stabilize and promote the RNA/protein interactions (Londei et al., 1986).

Notably, however, it was never possible to achieve *in vitro* reconstitution of functional *S. solfataricus* 30S subunits despite the lower complexity of these particles with respect to the 50S ones. More precisely, *in vitro* assembly of 30S particles containing the 16S rRNA and the whole complement of 30S ribosomal proteins was easily obtained, but they were not active in translation (Londei, unpublished). The reason for this unexpected result is unclear. It may be due to the substantially higher protein content of *S. solfataricus* 30S subunits with respect to bacterial particles (28 r-proteins vs 20–21), and/or to the requirement for some additional assembly-promoting factor (see below). If so, biogenesis of *S. solfataricus* 30S subunits may present interesting homologies with the eukaryotic process that would be worth exploring in better detail.

As to halophilic ribosomes, *Haloferax mediterranei* 30S and 50S subunits could be reassembled successfully only at very high concentrations of salt, close to the physiological concentration within the cell. Two types of monovalent cations were the most effective in promoting reconstitution, K^+^ and NH_4_^+^. Unlike what happens for both *E. coli* and *S. solfataricus, H. mediterranei* ribosomes could be reconstituted using a single-step incubation at 42°C., i.e., within the optimal temperature range for physiological growth of this organism. The procedure was similar for 30S and 50S subunits except that reconstitution of 30S subunits had a higher tolerance to ionic strength than that of 50S subunits and was independent of the Mg^2+^ concentration present in the assay (Sanchez et al., 1990, 1996).

One important outcome of the *in vitro* reconstitution experiments with archaeal ribosomes was the possibility of studying the assembly pathways and to identify the assembly-initiating r-proteins. Indeed, using purified rRNA and r-proteins from *S. solfataricus* large ribosomal subunits, it was shown that the initial RNA–protein interactions leading to the formation of a definite but still incomplete assembly intermediate did not require high temperatures, but took place optimally at about 20°C (Altamura et al., 1991). High temperatures, plus the missing proteins, were instead mandatory to convert the low-temperature assembly intermediate into active complete subunits. The assembly intermediate contains 16 of the 34 total 50S subunit r-proteins; among these, the actual primary RNA-binding proteins were identified by experiments of rRNA binding to membrane-immobilized *S. solfataricus* large subunit proteins. These turned out to be 8–9 r-proteins, well in accordance with the number of primary RNA-binding proteins in bacterial 50S ribosomes. It is probable that some, or even all, of these proteins belong to the universally conserved set of r-proteins, but because their identity was not assessed in the study in question, this cannot be stated with certainty. In any event, that the r-proteins present in the low-temperature-assembly intermediate are the innermost in the body of the 50S subunit was also confirmed by preparing ribosomal “cores,” i.e., stripping the outer r-proteins with high concentrations of LiCl, a salt known to disrupt weak RNA/protein interactions (Altamura et al., 1991).

Finally, the availability of methods for *in vitro* reconstitution of archaeal ribosomes allows exploring the degree of evolutionary conservation of the assembly pathways and of rRNA/r-protein interactions. In one study, it was found that incubation of *S. solfataricus* LSU proteins with the 23S rRNAs from a distantly related archaeon (*H. mediterranei*) or from *E. coli* led to the formation of a definite and compact 40S particle, containing most of the proteins previously identified as early assembly proteins in *S. solfataricus*, including all of the primary RNA-binding ones (Altamura et al., 1991). These results suggest that the basic architecture of the ribosome and the primary rRNA/r-protein interactions are conserved to a large extent in the two prokaryotic domains of life.

Other data in agreement with this surmise is the complete functional exchangeability of 5S rRNA between *S. solfataricus* and *E. coli* LSUs (Teixidò et al., 1989).

In contrast, incubation of the *S. solfataricus* whole complement of 50S ribosomal proteins with LSU rRNAs from yeast produced no particle, but only an heterogeneous array of RNP complexes, further indicating that both ribosome structure and assembly pathways have undergone a marked divergence from the prokaryotic model in the course of eukaryotic evolution (Altamura et al., 1991).

In summary, probably the most important lesson to be learned from the *in vitro* assembly experiment is that strong similarities exist in the basic architecture and assembly pathways of archaeal and bacterial ribosomes in spite of the presence of unique features in both and of certain “eukaryotic” features in archaea, especially as regards rRNA structure and maturation. The greater complexity of ribosome assembly in eukaryotes is best documented by the fact that, despite many efforts, *in vitro* reconstitution of functional eukaryotic ribosomes from the separated components was largely unsuccessful. The one study claiming success in this task was performed with the ribosomes of *Dictyostelium discoideum* (Mangiarotti and Chiaberge, 1997). Interestingly, *in vitro* assembly of functional *D. discoideum* ribosomes could not be achieved using 18S and 28S rRNA species from mature cytoplasmic ribosomes but required still immature rRNA extracted from nuclear ribosomes. Furthermore, a small RNA species—presumably nucleolar—is apparently required for successful reconstitution. Although this study was never replicated, it agrees with the fact that ribosome assembly is inherently more complex in eukaryotes, developing along a pathway that makes use of many additional extra-ribosomal nuclear/nucleolar factors. Also, the similarity in operon organization and in processing pathways of archaeal and bacterial rRNAs with respect to the eukaryotic ones speaks in favor of a greater evolutionary conservation between the two prokaryotic domains. The presence of a single cellular compartment in which everything happens, from transcription of rRNAs, to maturation of rRNA transcripts, to ribosome assembly and activation, must have dictated the need for a simpler and more streamlined process of ribosome biogenesis than it is the case for eukaryotes. However, more work is required to assess these points, especially *in vivo* experiments, which, at present, are almost completely lacking in archaea.

## Ribosome Biogenesis Factors: Archaeal Specificity and Shared Features

Ribosome biogenesis also requires the participation of additional ribosome biogenesis factors, also known as assembly factors or *trans*-acting factors. These factors have been analyzed in great detail in bacteria and eukaryotes. Generally, these factors transiently interact with the nascent ribosomal subunits and are believed to facilitate the ribosome biogenesis process. Among these factors, a significant fraction homes various enzymatic activity, mostly NTPase activity (ATPase, GTPase, and RNA helicases…), which may contribute to promote energy-dependent steps of the ribosomal subunit biogenesis process. Interestingly, whereas GTP-dependent processes are predominant in bacteria, ATP-dependent processes are strikingly more frequent in Eukaryotes (Shajani et al., 2011; Thomson et al., 2013; Davis and Williamson, 2017; Baßler and Hurt, 2019; Klinge and Woolford, 2019). Paradoxically, and despite the universal conservation of the ribosomal subunits, most of the ribosome biogenesis factors are (1) not conserved across evolution, and (2) their numbers are dramatically increasing in eukaryotes (Hage and Tollervey, 2004; Ebersberger et al., 2014; Ferreira-Cerca, 2017; Figures 1, 3). This observation suggests that the ribosome biogenesis pathway has been reengineered multiple times during evolution and may reflect early adaptation to molecular constraints present within the respective cellular lineage ancestors. Still, there are remarkable similarities and/or analogies between the different ribosome biogenesis pathways that may exist and are worth being highlighted. First, the presence of ribosome biogenesis factor sequence homologs between archaea and eukaryotes suggests a common origin of the archaeal–eukaryotic ribosome biogenesis pathway (Ebersberger et al., 2014). Intriguingly, these sequence homologs are known to predominantly act during the latest steps of eukaryotic SSU and LSU maturation. Second, the presence of structural and/or functional mimicry conserved across the tree of life suggests that, despite an apparent sequence/structure divergence between most ribosome biogenesis factors, some steps have similar molecular constraints across the tree of life that are overcome by functionally equivalent molecular inventions [discussed in Ferreira-Cerca (2017); Jüttner et al. (2020)]. This seems to be particularly true in the context of the late steps of the small ribosomal subunit biogenesis (Ferreira-Cerca, 2017; Knüppel et al., 2018). Notably, despite the absence of apparent sequence and structural conservation between bacterial and eukaryotic ribosome biogenesis factors, those, for example, involved in the late steps of SSU maturation remarkably cluster within an analogous structural region on the nascent SSU, i.e., regions that form the future functional centers. This suggests that binding of these ribosome biogenesis factors may ensure functional testing and avoid premature release of the nascent ribosomal subunits into the translational pool (Strunk et al., 2011, 2012; Ferreira-Cerca, 2017; Ghalei et al., 2017; Parker et al., 2019).

Furthermore, the ribosome biogenesis factors sequence homologs are not evenly distributed across all archaeal genomes, but follow the reductive evolution trend observed for the r-proteins, thereby suggesting a simplification of the ribosome biogenesis pathway in these organisms, e.g., euryarchaeota or nanoarchaeota, whereas ribosome synthesis in the TACK superphylum may generally be more complex due to the presence of additional r- proteins or ribosome biogenesis factors (Lecompte et al., 2002; Yutin et al., 2012; Ebersberger et al., 2014; Zaremba-Niedzwiedzka et al., 2017; Figures 1, 3). However, in organisms showing an apparent reduced ribosome biogenesis complexity, the addition or molecular exchange by unknown archaeal specific r-proteins and/or ribosome biogenesis factors cannot be fully excluded.

So far, the functional analysis of archaeal ribosome biogenesis factors is rather limited, and only a few have been established *in vivo*. Most of these characterized factors are sequence homologs of genuine eukaryotic ribosome biogenesis factors [see Ebersberger et al. (2014) for a complete list of candidates]. Among them, the dimethyltransferase KsgA/Dim1 [see above and Grünberger et al. (2020); Knüppel et al. (2021)], or the Rio ATPase/Kinase family members are implicated in the late steps of SSU maturation, where they probably play a role similar to their eukaryotic counterparts (Knüppel et al., 2018). Similarly, the endonuclease Nob1 is implicated in the maturation of the 16S rRNA 3′end *in vitro* (Veith et al., 2012; Qi et al., 2020; Figure 2). Collectively, these analyses suggest that the late steps of archaeal SSU biogenesis is a simplified version of the late steps of eukaryotes SSU maturation (Ferreira-Cerca, 2017; Knüppel et al., 2018). However, the degree of functional conservation and interactions of ribosome biogenesis factors such as the archaeal homologs of Rio1, Fap7, Dim1, Pno1, or Nob1, which form an important functional network involved in the late steps of eukaryotic SSU maturation, remains to be explored. Gaining information on these points will surely offer important insights on the molecular evolution and adaptation of the ribosome biogenesis pathway.

Last, the endonuclease *endA* known to be involved in the maturation of intron-containing tRNAs (Clouet-d’Orval et al., 2018) has been recently implicated in rRNA processing, thereby indicating a functional coordination of tRNA and rRNA maturation in archaea (Qi et al., 2020; Schwarz et al., 2020; Figure 2).

## Perspectives and Outlook

Among the numerous challenges and outstanding questions ahead, the comprehensive identification and functional characterization of factors implicated in archaeal ribosome biogenesis are a key step to further understanding the common and specific features of archaeal ribosome biogenesis. In addition, recent improvement of cryo-electron microscopy analysis has been instrumental to better characterize bacterial and eukaryotic ribosome biogenesis pathways (Davis and Williamson, 2017; Baßler and Hurt, 2019; Klinge and Woolford, 2019). A similar revolution is still to come in the archaeal ribosome biogenesis field and will be important to decipher functional and structural analogies conserved across the tree of life and further improve our view on the evolutionary history of the ribosome biogenesis pathway and how molecular and environmental constraints may have (re-)shaped the ribosome biogenesis molecular dance.

Furthermore, and as discussed, the ribosome biogenesis sequence homologs and r-proteins are not ubiquitously distributed across archaea. Therefore, it is of interest to define the extent of archaeal ribosome biogenesis diversity and functional adaptation (Seistrup et al., 2017; Birkedal et al., 2020; Sas-Chen et al., 2020; Knüppel et al., 2021). Additionally, future metagenomics analyses will certainly increase the numbers of newly identified archaea. Accordingly, learning from archaeal biodiversity, changes and adaptation of the ribosome biogenesis pathway are expected to be discovered; however, the formal analysis of this biodiversity is only possible with the advance of culturomics (Bilen et al., 2018; Lewis et al., 2021) and the fast implementation of genetic manipulation in multiple archaeal organisms.

## Author Contributions

PL and SF-C wrote the manuscript. Both authors contributed to the article and approved the submitted version.

## Conflict of Interest

The authors declare that the research was conducted in the absence of any commercial or financial relationships that could be construed as a potential conflict of interest.
